# An unusual occurrence of Kleine-Levin syndrome in a man with refractory immune thrombocytopenic purpura: a case report

**DOI:** 10.1186/s13256-015-0536-5

**Published:** 2015-04-01

**Authors:** Hamed Amirifard, Farzaneh Barzkar, Seyed Amirhossein Fazeli, Seyed Mehdi Hashemi

**Affiliations:** 1 Department of Neurology, Zahedan University of Medical Sciences, Zahedan, Iran; 2 Students’ Scientific Research Center, Zahedan University of Medical Sciences, Zahedan, Iran; 3 Department of Internal Medicine, School of Medicine, Zahedan University of Medical Sciences, Zahedan, Iran; 4 Department of Hematology, Zahedan University of Medical Sciences, Zahedan, Iran; 5 Ali-Ebne-Abitaleb hospital, Zahedan, Iran

**Keywords:** Autoimmunities, Excessive somnolence disorder, Idiopathic (immune) thrombocytopenic purpura, Kleine-Levin syndrome, Meningococcal vaccine, Refractory

## Abstract

**Introduction:**

Kleine-Levin syndrome is an extremely rare neurological entity characterized by recurrent episodes of hypersomnia which are sometimes associated with compulsive hyperphagia and behavioral changes. Autoimmunity has recently been proposed as a factor contributing to its pathogenesis. Immune thrombocytopenic purpura is a relatively common autoimmune disease showing a lot of complexity and uncertainty regarding its treatment regimens and its refractory nature in some cases.

**Case presentation:**

A 32-year-old Persian White man visited his private hematologist complaining of recent episodes of epistaxis and appearance of petechial lesions 24 hours after receiving a meningococcal vaccine. He had a history of immune thrombocytopenic purpura 13 years before his presentation. Based on his history and laboratory findings, his condition was diagnosed as a relapse of immune thrombocytopenic purpura and was managed accordingly. He did not respond to first-line corticosteroid regimens and later developed neurological symptoms as recurrent episodes of hypersomnia and hyperphagia. After a complete clinical and paraclinical evaluation and ruling out other possible conditions, he was given a diagnosis of Kleine-Levin syndrome. He was followed up for his immune thrombocytopenic purpura and received different treatment regimens none of which were adequately successful except intravenous immunoglobulin that was only temporarily effective. He has had 4 documented self-limited episodes of Kleine-Levin syndrome since his initial presentation.

**Conclusions:**

Immune thrombocytopenic purpura may be associated with meningococcal vaccination in adulthood. Responses to treatment in immune thrombocytopenic purpura vary among patients. Our patient only had a transient acceptable response to intravenous immunoglobulin while all other options failed to improve his platelet count. Concurrence of immune thrombocytopenic purpura and Kleine-Levin syndrome supports the role of autoimmunity as the proposed pathophysiological mechanism of Kleine-Levin syndrome.

## Introduction

Kleine-Levin syndrome (KLS) is a very rare neurological disorder characterized by relapsing-remitting periods of hypersomnia which are sometimes associated with compulsive hyperphagia and abnormal behavioral changes. Although mainly affecting male adolescents, the disease has been reported among people at a range of 4 to 82 years of age [1,2]. No population-based studies are available regarding its prevalence. Nevertheless, 186 cases of KLS have been reported worldwide according to the latest systematic review of cases published in 2005 only three of which were from Iran. The factors triggering the onset of the disease remain to be elucidated, while a number of precipitating factors have been reported in the literature the most common of which is a preceding infection such as a flu-like illness, an upper respiratory tract infection, a nonspecific fever, or a summer gastroenteritis with the symptoms occurring 3 to 5 days following the onset of fever or infection. Its occurrence has also been reported following consumption of alcohol or marijuana for the first time or in large amounts [3]. There are some hypotheses explaining the pathogenesis of KLS one of which is a post-infectious and autoimmune reaction affecting the thalamus, hypothalamus, and frontal, temporal, or occipital lobes of the brain [3-5]. Some researchers have recently focused on the autoimmune factors underlying the pathogenesis of KLS including specific associated human leukocyte antigen (HLA) types [6].

Immune thrombocytopenic purpura (ITP) is a relatively common autoimmune hematological disorder with a predilection for young women over men. Its pathogenesis involves both platelet destruction and impaired platelet production both of which are mediated by the immune system [7,8]. It can occur in isolation or with other autoimmune diseases such as systemic lupus erythematosus. It has also been reported to occur following the administration of certain drugs and vaccines [9-11]. The goal of the treatment is to increase the platelet count to prevent bleeding events associated with thrombocytopenia [7]. Treatment with immunomodulatory agents remains the main component of the treatment although many cases have recently been refractory to such drug regimens [12]. A majority of children with ITP recover spontaneously while in adults it follows a more persistent course and often requires more rigorous medical interventions [13].

In this case report we describe a 32-year-old man with a chronic refractory ITP who developed symptoms suggestive of KLS early in the course of ITP. This concurrence of diseases together with the autoimmune background of ITP and the factors inducing their initiation may introduce areas of further research on the pathophysiological background of KLS. Moreover the occurrence of KLS at this age is exceptionally rare according to the literature [6,14]. This case has many unique educational aspects regarding the course of ITP including the triggers that can potentially play a role in its relapse.

## Case presentation

**Figure 1:**
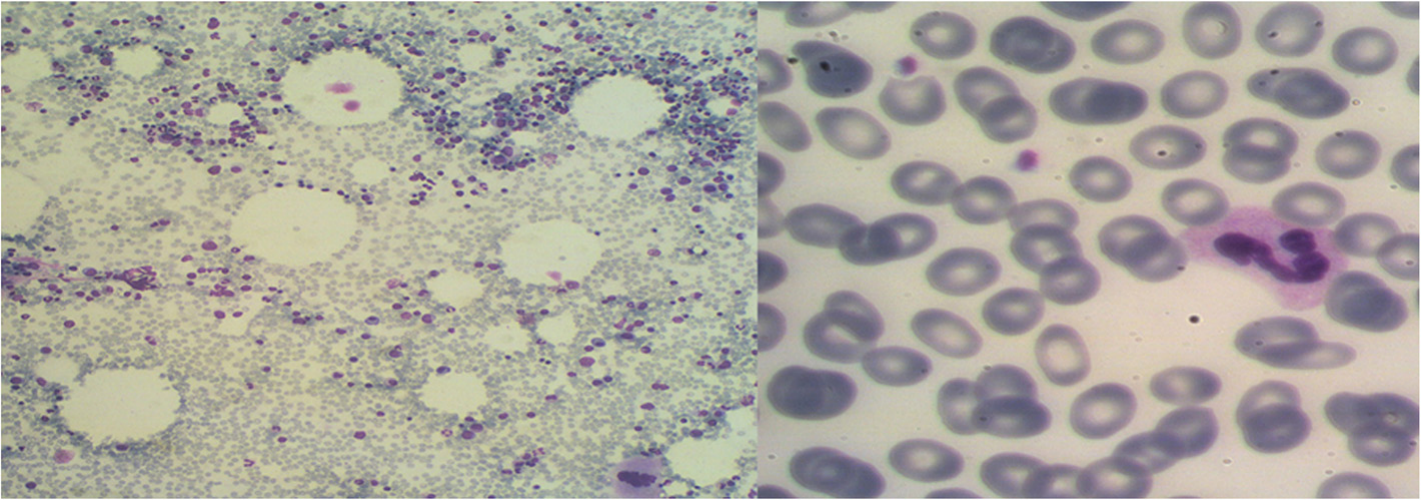
**Bone marrow biopsy and peripheral blood smear.** Low number of platelets without clumping, normal megakaryocytes and normal leukocyte and erythrocyte number and morphology suggest immune thrombocytopenic purpura.

A 32-year-old Sistani-Persian White man presented to a hematologist for the recent appearance of petechial lesions and epistaxis. He mentioned a history of ITP 13 years prior to his visit which had not responded well to standard corticosteroid therapy, was managed with splenectomy after 3 months of resistance to steroids and had remitted since that time until the recent reappearance of symptoms. He also reported having had pharyngitis 2 weeks before his current symptoms onset that had not been improved by amoxicillin-clavulanate administration. He also reported a history of recent meningococcal vaccination and stated that his symptoms had started within 24 hours after meningococcal vaccination. The laboratory results showed a platelet level of 25,000 per microliter. With a diagnosis of relapsing ITP, he was started on standard prednisolone regimen for 1 month which did not improve his condition. After 2 months he was seen by another hematologist for further evaluation and persistence of thrombocytopenia. A bone marrow biopsy was performed which confirmed the diagnosis of ITP (Figure 1). He also underwent a scan for accessory spleen which was negative. Following this visit, he was given 40mg/day of pulsed dexamethasone for 4 days every 2 weeks for 8 weeks in addition to pulsed rituximab (800U/week) for 4 cycles neither of which was sufficiently effective to reduce his symptoms. Detailed information regarding his medication history following this visit is presented in Table 1.

**Table 1:** **Medication history**

**Date of visit**	**Medication**	**Dosage and route**	**Duration of treatment**	**Response to treatment/concurrent events**
April, year 1	Prednisolone	80mg/day orally	1 month	No response
May, year 1	Rituximab	800U/week	4 weeks	No response
	Dexamethasone	40mg/day pulsed	4 days/week every two weeks up to four cycles	No response
July, year 1	Azathioprine	100mg/day	3 months	No response
	Danazol	200mg/three times a day, orally	3 months	No response
October, year 1	Previous regimen + Cellecept®(mycophenolate mofetil)	1g/three times a day	4 months	No response
February, year 2	Endoxan® (cyclophosphamide)	1g/ every 3 weeks	(four cycles)	No response
April, year 2	Colchicine	1mg/day	2 months	No response
	Chloroquine	1 tablet twice a day	2 months	No response
June, year 2	Danazol	200mg/three times a day	3 months	No response
	Prednisolone	50mg Every other day	3 months	Continued to present as maintenances therapy
September, year 2	Intravenous immunoglobulin	1g/kg/day for 2 days	Four cycles	Platelet elevation to 300,000 for 2 weeks
January, year 3	Sirolimus	Day 1: 6mg Maintenance dose: 2mg/day	1 month	First neurological attack
February, year 3	Intravenous immunoglobulin	1g/kg/day	For 2 days 3 cycles	Transient improvement of platelet count
March, year 3	Sirolimus	2mg/d	11 months	No response
	Oncovin®(vincristine)	2mg/week intravenously	For 2 weeks	Side effects: paresthesias, severe abdominal pain
April, year 3	Sandimmune® (cyclosporine)	900mg orally/day	–	Intolerance
	Chlorambucil	14mg/day for 28 days	Two cycles	No response

Twenty months after relapse of ITP, when our patient was taking sirolimus, he presented to our hematology clinic again with sudden onset of new symptoms including hypersomnia, abnormal behavioral changes, clumsiness, and compulsive hyperphagia. His wife reported that her husband slept approximately 20 hours a day during his symptomatic period, did not wake up spontaneously except for voiding and could not be awakened upon calling on him. She also recounted that her husband had drunk coffee the night before his onset of symptoms. Computed tomography (CT) scanning and magnetic resonance imaging (MRI) of his brain and spinal cord were unremarkable; his symptoms improved spontaneously, thus he was discharged. It continued this way until recurrence of neurologic symptoms 3 months later in temporal association with a thrombocytopenic attack with a platelet count of 12,000. During these episodes, he was hospitalized and given two doses of intravenous immunoglobulin (IVIG) and steroids resulting in a temporary improvement. He was referred to our neurology department for consultation.

On evaluation at our neurology department, he was amnesic about details of events during his symptomatic periods. His wife though described the last attack as around 18 to 20 hours of daily sleep during which time he woke up spontaneously two to three times and could be awakened by calling on him. She also reported excessive food intake by her husband (seven to eight complete meals a day) during the recent episode with a much stronger tendency for sweet foods. She stated that her husband had delayed ejaculation during his sexual intercourse and mentioned the length of each episode of neurological symptoms as one to two weeks.

His medical history apart from ITP was only significant for malaria during his childhood which had been managed successfully. He denied any history of narcolepsy, sleep disorders, or seizures. He is a Persian White married man with one healthy son and lives in an urban area in southeast of Iran. He did not use any illicit drugs, drink alcohol or smoke cigarettes. His detailed medication history is given in Table 1. His family history is not significant for any neurological illnesses or sleep disorders. His mother had an aortic incompetence which was treated with surgery at the age of 45.

On physical examination, he was not ill or toxic and appeared his stated age. His vital signs, head and neck, chest, abdomen, genitalia, and extremities were normal. He was alert, oriented to time and place, with mild psychomotor slowness. His cranial nerves were intact and motor tone, force and reflexes were normal. Pinprick, light touch and vibration were intact. However, he was found to have bilateral bidirectional nystagmus with central pattern in the Hallpike test. He had an otherwise normal gait apart from clumsiness and general slowness.

The differential diagnosis included episodic ataxia, mood disorders, and metabolic encephalopathy including abnormalities of urea-ammonia cycle, Lyme disease, Klüver–Bucy syndrome, and KLS.

**Figure 2:**
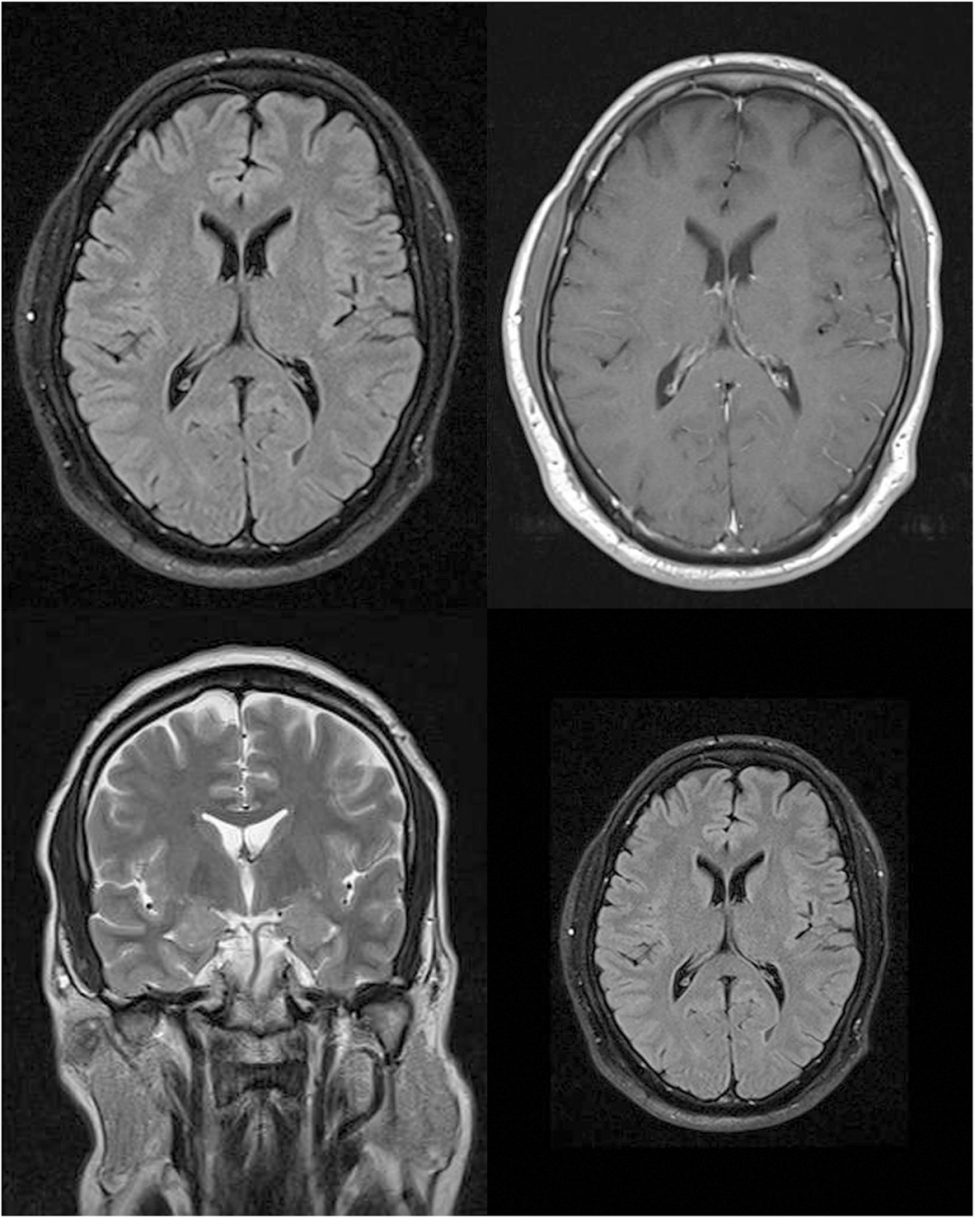
**Brain magnetic resonance imaging images.**

A complete work-up including lumbar puncture was conducted. Laboratory data including cerebrospinal fluid study and liver and renal function tests were not significant by any means except for findings related to the ITP which was a platelet level of 25,000 per microliter and a mild vitamin B12 deficiency. MRI and CT scans were normal with no space-occupying lesions or signs of inflammatory lesions (Figure 2). Electroencephalography did not show any notable pathological changes.

He experienced another episode a few months later with similar symptoms although less severely with 12 to 14 hours of sleep every day and milder hyperphagia and sexual disturbances but with nystagmus upon speaking and a lag in gaze control. This resolved spontaneously during the course of a few days as well.

After 2 months, he was referred to a referral hospital in another city with single-photon emission computed tomography (SPECT) facilities for evaluation of brain perfusion. He was given 40mg of dexamethasone daily prophylactically 2 days before his flight. While boarding the plane, he started feeling dizzy and had mild imbalance and an abnormal gait. On the plane he slept the whole length of the flight much to the surprise of his wife as usually he never managed to sleep on a plane. He was only awakened by his wife for dinner at which time he ate both his own and his wife’s meals completely and slept again immediately. He did not even wake up after landing in order to get off the plane. He got better after he left the plane and recovered over a course of 3 to 4 days when he underwent SPECT. SPECT results were also completely normal and did not show anything in favor of any specific diagnoses.

He was given a diagnosis of KLS supported by his normal laboratory and imaging studies and after ruling out the above-mentioned conditions. However he was not given any specific therapy for this diagnosis as he was not concerned about the impact of the episodic symptoms on his quality of life. His platelet counts remain below 10,000/μL having only temporary responses to IVIG in the acute setting.

## Discussion

Relapse of ITP after splenectomy is a relatively rare phenomenon involving less than 10% of patients initially diagnosed with ITP which is scientifically categorized as a refractory form of the disease [15]. Thrombocytopenia may occur with an incidence of 1 in 25,000 to 40,000 vaccinations for measles, mumps, and rubella, and more rarely after vaccination against pneumococcus, *Haemophilus influenzae* B, varicella zoster virus, and hepatitis B, less than 10% of which evolve into chronic ITP [16]. Our patient’s symptoms appeared within hours after meningococcal vaccination. Although this does not necessarily indicate an association between meningococcal vaccination and relapse of ITP as the pharyngitis was probably the main culprit, to the best of our knowledge, this concurrence has not been reported previously in the literature and is therefore of considerable value.

Idiopathic (immune) thrombocytopenic purpura is defined as isolated thrombocytopenia for which other causes have been ruled out. There are no established criteria for its diagnosis. The American Society of Hematology released a guideline in 2011 giving evidence-based recommendations aiding the diagnosis and management of ITP. This guideline was largely based on the International Working Group consensus panel [17]. These guidelines no longer consider bone marrow examination a necessary diagnostic measure and only recommend it in certain circumstances; instead, they suggest the diagnosis be based on findings obtained from patient’s medical history, physical examination, complete blood count, and peripheral blood smear. There is also insufficient evidence supporting the use of laboratory tests such as antiplatelet antibodies, antiphospholipid antibodies, and antinuclear antibodies for initial diagnosis of ITP [18]. Our patient had typical signs of thrombocytopenia, a positive past medical history of severe ITP, a persistent platelet count of ≤30×10^9^/L, and a blood smear with suggestive findings, which fulfills the definition of refractory ITP according to the guidelines which define it as a severe ITP relapsing after splenectomy. We also performed a bone marrow examination as the patient had no response to usual recommended medications which was consistent with the diagnosis of ITP as well. Moreover, a transient response to IVIG by itself prompts the immune pathogenesis of the underlying condition and further verifies the diagnosis of ITP.

Treatment of ITP is only indicated in symptomatic patients with platelet counts <30 to 50×10^9^/L.First-line therapy consists of corticosteroids, IVIG, and anti-D antibodies. In patients unresponsive to these therapeutic options, splenectomy may be indicated. Splenectomy is considered the most efficacious treatment with 60 to 86% of patients going into complete remission postoperatively. A patient who does not respond to the first two lines of therapy (25 to 30% of all patients with ITP) and remains symptomatic for more than 1 year, as applies to our patient, is classified as having chronic refractory ITP. There is a predicament whether treatment should be continued and is of acceptable benefit in this group of patients as current third-line drugs are mostly immunosuppressive and may put the patients at risk of severe infections [12,17]. Our patient fell into this group and considering his symptoms and concerns and weighing out the risks against benefits, we initiated the treatments as presented in detail in Table 1. However, our patient did not respond to the therapies suggested by the present evidence, including immunosuppressive and chemotherapeutic agents, except for a short-term response to IVIG that made IVIG a useful option in combination with corticosteroid pulse therapy in the management of ITP in the emergency setting. This finding is also supported by the literature, which indicates that IVIG is at least transiently useful for emergency management even in patients with chronic refractory ITP [12,17,18]. IVIG has also been shown to be effective in other diseases of an autoimmune nature, one of the earliest being its use in Guillain–Barré syndrome and in our case was also effective in reducing symptoms of KLS[19]. The new guidelines, however, also recommend the use of thrombopoietin receptor agonists as one of the options for treatment failure after splenectomy, which we did not have access to in our center [18].

KLS is an exceptionally rare neurological disorder characterized by recurrent episodes of hypersomnia which primarily affects adolescents. Our patient experienced the symptoms at the age of 32 and this is considered an even more unusual phenomenon although the disorder has also been reported at older ages [1,20]. The exact pathogenesis of the disease is unknown as most of the clinical and paraclinical findings are usually normal. However, hypoperfusion and other nonspecific abnormalities in the left hypothalamus, thalamus, basal ganglia, medial and dorsolateral frontal and temporal regions have been reported [4,21,22]. In the past 2 decades, autoimmune mechanisms have been proposed as the culprits participating in its pathogenesis. HLA-DQB1 has been reported to be associated with the disease in a controlled study on 30 patients with the syndrome [5], although these results have not been repeated in later studies [6]. Our patient developed KLS against a background of an autoimmune disorder. This finding may highlight the role of immunity in the pathogenesis of this rare syndrome [1,14].

Of note, we had limitations on doing some of the relevant paraclinical studies such as polysomnography, nuclear imaging during the episodes, and HLA typing due to scarcity of resources and facilities at our center.

## Conclusions

Our findings and other reported associations of some vaccines with onset of ITP warrant the need for further better quality research in this field and weighing out their risks against their benefits in order to reach a consensus on their risks and whether such vaccines should be used more cautiously in patients more prone to ITP.

This presented case of refractory ITP suggests that currently available treatments are not sufficient and that new drugs such as thrombopoietin agonists may turn out to be life-saving in such chronic refractory patients.

## Consent

Written informed consent was obtained from the patient for publication of this case report and any accompanying images. A copy of the written consent is available for review by the Editor-in-Chief of this journal.

## Abbreviations

CT: Computed tomography
HLA: Human leukocyte antigen
ITP: Immune thrombocytopenic purpura
IVIG: Intravenous immunoglobulin
KLS: Kleine-Levin syndrome
MRI: Magnetic resonance imaging
SPECT: Single-photon emission computed tomography
